# Challenges in Adapting Fibre Optic Sensors for Biomedical Applications

**DOI:** 10.3390/bios15050312

**Published:** 2025-05-13

**Authors:** Sahar Karimian, Muhammad Mahmood Ali, Marion McAfee, Waqas Saleem, Dineshbabu Duraibabu, Sanober Farheen Memon, Elfed Lewis

**Affiliations:** 1 Centre for Mathematical Modelling and Intelligent Systems for Health and Environment (MISHE), Atlantic Technological University, F91 YW50 Sligo, Ireland; muhammad.ali@atu.ie (M.M.A.); marion.mcafee@atu.ie (M.M.); dineshbabu.duraibabu@atu.ie (D.D.); 2 Department of Mechatronic Engineering, Faculty of Engineering and Design, Atlantic Technological University, F91 YW50 Sligo, Ireland; 3 Department of Mechanical Engineering, Technological University Dublin, D15 YV78 Dublin, Ireland; waqas.saleem@tudublin.ie; 4 Optical Fibre Sensors Research Centre, University of Limerick, V94 T9PX Limerick, Ireland

**Keywords:** fibre optic sensors, biomedical applications, biocompatibility, glucose measurement, signal processing

## Abstract

Fibre optic sensors (FOSs) have developed as a transformative technology in healthcare, often offering unparalleled accuracy and sensitivity in monitoring various physiological and biochemical parameters. Their applications range from tracking vital signs to guiding minimally invasive surgeries, enabling advancements in medical diagnostics and treatment. However, the integration of FOSs into biomedical applications faces numerous challenges. This article describes some challenges for adopting FOSs for biomedical purposes, exploring technical and practical obstacles, and examining innovative solutions. Significant challenges include biocompatibility, miniaturization, addressing signal processing complexities, and meeting regulatory standards. By outlining solutions to the stated challenges, it is intended that this article provides a better understanding of FOS technologies in biomedical settings and their implementation. A broader appreciation of the technology, offered in this article, enhances patient care and improved medical outcomes.

## 1. Introduction

As sensor technology advances, Fibre Optic Sensors (FOSs) have gained prominence for their accuracy, high sensitivity, and resistance to electromagnetic interference. These characteristics make them particularly attractive for biomedical applications, where accurate and reliable measurements are crucial [1,2]. From monitoring physiological parameters to aiding in minimally invasive surgeries, fibre optic sensors hold the potential to revolutionize medical diagnostics and treatment [3].

The adoption of fibre optic sensors in the biomedical field requires several obstacles to be overcome. Integrating these sensors into medical devices requires overcoming challenges related to biocompatibility, miniaturization, and effective and efficient signal processing. Additionally, ensuring the robustness, real-time responsivity, and reliability of FOSs in the dynamic and often harsh environments of the human body presents another layer of complexity. Also, their use requires meeting international standards, which can also be challenging in the pathway to commercialization [4,5].

**Figure 1:**
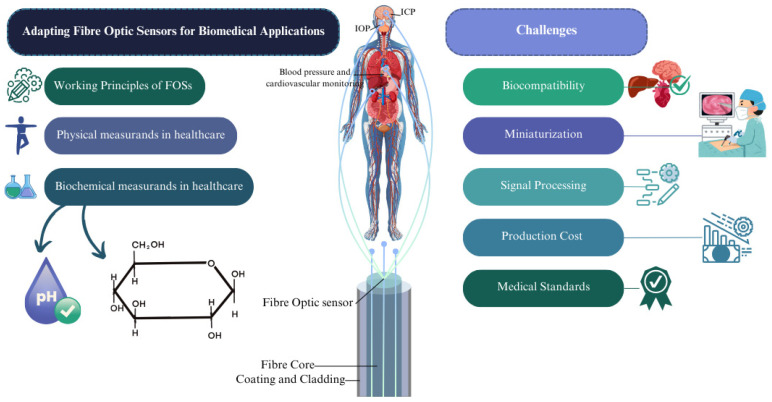
An abstract of the review, showing challenges regarding adoption of FOSs in real-world applications.

This review explores the key challenges encountered in adopting fibre optic sensors for biomedical applications. It presents the background and working principles of these sensors, along with an assessment of key physical and biochemical measurands. The review then examines the challenges associated with FOS implementation, focusing on issues such as biocompatibility, miniaturization, signal processing, manufacturing costs, various medical standards, and ethical considerations relevant to biomedical use. In the final section, the conclusion highlights potential future directions for fibre optic sensors in both the scientific community and real-world applications. Figure 1 provides an abstract overview of the review, highlighting the key challenges associated with the adoption of FOSs in real-world applications, based on different physical and biochemical measurands.

### 1.1. Background

FOSs transmit light through optical fibres, where changes in properties such as intensity, phase, or wavelength indicate specific physiological conditions. In the biomedical field, FOSs enable the real-time monitoring of vital signs, including pressure and temperature; facilitate biochemical detection; and support minimally invasive surgical procedures [6]. Despite their advantages, several challenges hinder their widespread adoption in healthcare. Key issues include ensuring biocompatibility, scaling manufacturing to meet industry standards, and developing efficient real-time signal processing algorithms. Overcoming these barriers is critical for integrating FOS technology into routine medical diagnostics and treatment, where it holds the potential to enhance accuracy and reliability in patient care [7].

**Figure 2:**
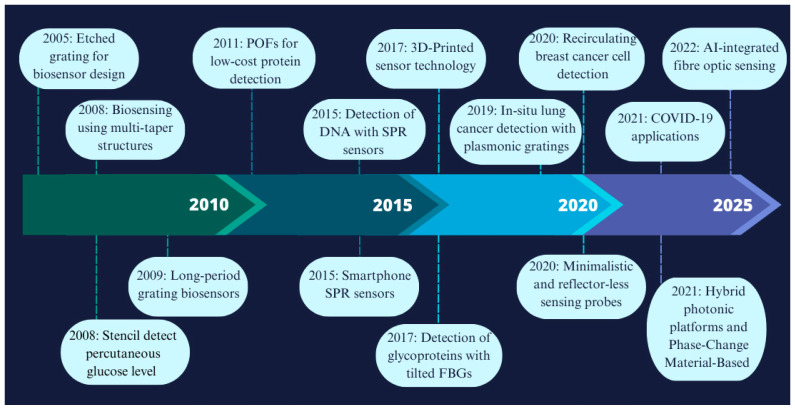
A chronology of some of the main achievements in fibre optic biosensor technology [9,10,11,12,13].

The origins of biosensing technology trace back to 1956, when Leland C. Clark Jr. (referred to as the “father of biosensors”) developed the first true biosensor for oxygen detection. A breakthrough came in 1962 when Clark introduced the first glucose biosensor, a technology that remains widely used and is continually evolving. Initially based on the detection of hydrogen peroxide, glucose biosensors have since progressed from basic laboratory demonstrations to commercialized devices, and now to advanced wearable and non-invasive systems for real-time monitoring [8]. Figure 2 illustrates some of the main achievements in fibre optic biosensor technology since 2005 and highlights the most significant milestones in the field [9].

**Figure 3:**
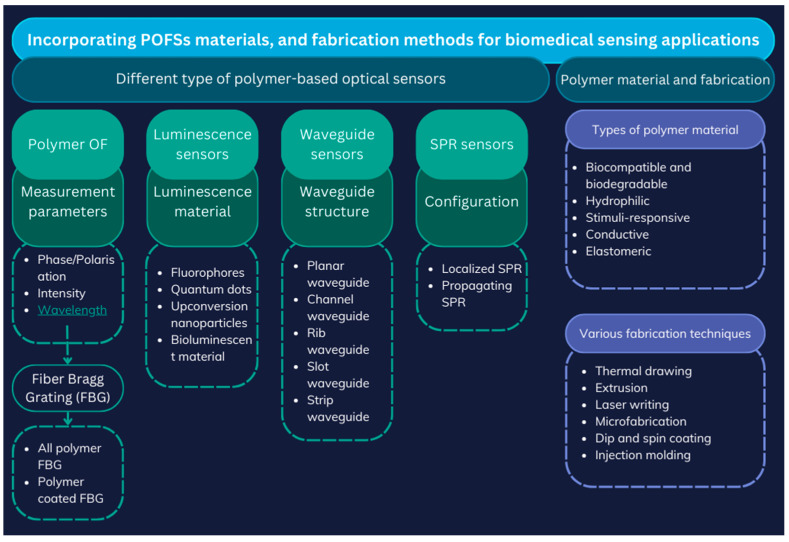
Different types of polymer-based FOSs with fabrication techniques; adopted idea: [22].

Polymer-based (or plastic) optical fibre sensors (POFSs) represent a significant advancement in FOS technology, offering flexible and adaptable sensing solutions. These sensors can operate based on various principles, including waveguide-based [14,15] mechanisms, luminescence [16], Surface Plasmon Resonance (SPR) [17], and optical fibre-based sensing, and they are suitable for both single-point and multi-point applications [18,19,20]. A particular focus is placed on polymer optical fibre sensors, due to their applications in biosensing. Polymeric materials, such as biodegradable, biocompatible, hydrophilic, stimuli-responsive, conductive, and molecularly imprinted polymers, further enhance biomedical sensing capabilities [21]. Additionally, various fabrication techniques for polymer optical fibres (POFs), including thermal drawing, extrusion, laser writing, microfabrication, and advanced coatings, are included in Figure 3 as classifications of different types of fibres and fabrication techniques [3,22,23].

### 1.2. Working Principles of FOSs

**Figure 4:**
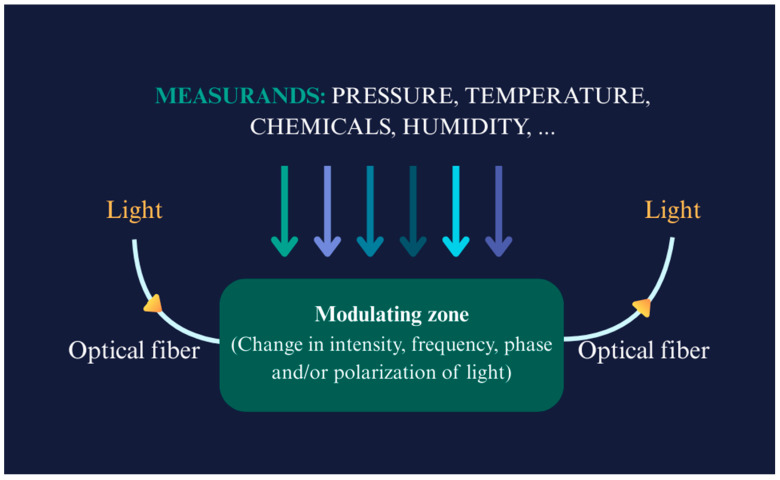
Working principles of a fibre optic sensor with different measurands (showed in arrows with different colours); adopted idea: [40].

FOSs vary widely in their physical appearances and characteristics, and their operating principles are correspondingly diverse. Primary FOS classification splits into intrinsic and extrinsic sensors [18]. Intrinsic FOSs rely on light–matter interaction occurring wholly within the fibre itself, where changes in light properties (intensity, phase, polarization, or wavelength) occur due to external influences such as temperature, pressure, chemicals, humidity, or strain. On the other hand, extrinsic fibre optic sensors use the optical fibre merely to transmit light to and from an external sensing element. Fibre Bragg Gratings (FBGs) [24,25] have represented a revolution in sensing using optical fibres. Their working principle and reflection of specific light wavelengths or shifts in response to different measurands is shown in Figure 4. Interferometric sensors (of which the FBG is an example) generally measure phase changes in light caused by external perturbations [4,26,27]. The FBGs’ interaction with light as it passes through the grating planes depends on the Bragg condition, and the first-order Bragg condition can be stated as shown below:

(1) $$\lambda_{B}=2n_{eff}\Lambda$$

where $\;n_{eff\;}$ denotes the effective refractive index, while Λ represents the grating period, and $\lambda_{B}\;$ stands for the Bragg wavelength.

Several FOS and polymer-based optical fibre (POF) sensor working principles cannot be included in this article owing to page length restrictions; e.g., fluorescence-based sensors [28,29] detect variations in fluorescence emitted by certain materials subjected to external light excitation and are often used for biochemical measurements. Other types of FOSs include interferometric-based devices such as Fabry–Pérot [26,30], Surface Plasmon Resonance (SPR) [31,32], and Optical Coherence Tomography (OCT) [33,34] devices, utilized in imaging applications and often incorporating specialist fibre, e.g., Photonic Crystal Fibre (PCF) [28,35]. They all leverage the interaction between light and the external environment to provide sensitive and accurate measurements in a wide range of applications [27,36,37,38,39]. They can be categorized in various ways, including fibre type, application, and sensing mechanisms, as outlined in Table 1.

**Table 1:** Recently introduced FOSs based on fibre type and their applications, with assessment on their sensing mechanisms.

Fibre Type	Application	Sensitivity	Sensing Mechanism	Ref.
SMF	Pressure	263.15 pm/kPa	FPI	[41]
OF	Pressure (IOP)	Low baseline drift (<2.8 mmHg) over >4.5 years	FPI with OCT	[34]
MMF	Pressure	2.49 nm/kPa	Interference-based sensing	[42]
OF	Pressure/temperature	55.468 nm/MPa (pressure), 0.01859 nm/°C (temperature)	FPI with MEMs	[43]
U-shaped MMF	Biosensing	1251.44 nm/RIU	LSPR	[44]
PC fibre	Biosensing	12,000 nm/RIU and 16,000 nm/RIU	SPR	[45]
D-shaped OF	Biosensing	5161 nm/RIU	SPR	[46]
D-shaped OF	Biosensing	4122 nm/RIU	LMR	[47]
D-shaped PC fibre	Biosensing	21,700 nm/RIU	SPR	[48]
D-shaped PC fibre	Biosensing	20,000 nm/RIU	SPR	[49]
Plastic OF	Cholesterol detection	140 mg/dL to 250 nm/dL	-	[50]
SMF	Temperature	210.25 KHz/°C	Vernier effect	[51]
Fibre tip integrated ZnO-nanowire-nanograting	Temperature	0.066 nW/°C	Bragg reflection	[52]
MMF with spherical end	Pressure/temperature	0.139 mV/kPa (pressure), 0.87 mV/°C (temperature)	RI modulation using MEMS-based silicon	[53]
SMF with a Hollow Silica Tube (HST)	Pressure	396 pm/kPa	FPI	[54]
SMF with FBG	Pressure	1.466 pm/kPa	FBG array	[55]
Ultra-miniature fibre optic sensor	Pressure (IPP)	(r ≥ 0.7, *p* < 0.001)	Diaphragm-based FO integrated with a proportional–integral–derivative (PID)	[56]
Distributed OF	Pressure	65.920 μϵ/kPa	Axial strain change detection with a sensitizing structure	[57]

Also, fibre optic sensors (FOSs) can be classified, based on their measurands, into physical and biochemical types. Physical FOSs detect pressure, temperature, and strain by analysing optical signal changes. Biochemical FOSs identify specific analytes, such as glucose or pH, e.g., using functionalized coatings. This classification highlights their versatility in biomedical and industrial applications. This review focuses on their applications in biomedical sensing.

### 1.3. Physical Measurands in Healthcare

FOSs are well suited for providing measurements of various physical measurands and are becoming increasingly accepted for patient monitoring and diagnostics. These measurands include temperature, pressure, strain, flow, liquid level, displacement, vibration, rotation, radiation, and biochemical markers. For example, body temperature measurements are vital for tracking temperature fluctuations during surgeries, post-operative care, and critical care settings, ensuring patient stability and the early detection of infections [58,59]. Pressure sensors are extensively used to monitor several medical pressure parameters, including blood pressure, Intracranial Pressure (ICP) [60,61], and Intraocular Pressure (IOP) [62,63], which are critical for managing conditions including hypertension, Traumatic Brain Injuries (TBIs) [64], and glaucoma [63]. Strain sensors are employed to monitor respiration by measuring chest wall movements, offering valuable data for respiratory therapy [4] and sleep studies, and managing conditions including asthma or chronic obstructive pulmonary disease (COPD) [5,65]. By providing real-time data, fibre optic sensors offer enhanced clinical decision making and improved patient outcomes, and contribute to the advancement of personalized medicine [66,67].

Biomechanical measures encompass the physical parameters of the human body, focusing on physical structure and movement, and accurate measurement is crucial for the successful monitoring of various parameters. FOSs can measure strain and deformation in tissues and organs, providing critical data for orthopaedic and rehabilitation applications [1,3]. For instance, it is possible to monitor the stress and strain on bones and joints during physical activities, aiding in the assessment and treatment of musculoskeletal disorders. Additionally, they have been used for posture monitoring and ulcer formation detection in patients who are required to use a wheelchair [68]. Furthermore, these sensors are employed in developing prosthetics and wearable devices, providing real-time feedback on mechanical performance and interaction with the body. By measuring these biomechanical parameters, FOSs support the diagnosis, treatment, and rehabilitation of various conditions and advance the field of biomechanics in healthcare [5,69,70,71,72,73].

### 1.4. Biochemical Measurands in Healthcare

All biochemical measurands could be considered vital parameters in healthcare, providing essential information about patients’ whole physiological and metabolic states. FOSs are increasingly used to measure these biochemical markers with high sensitivity and specificity. One significant application of FOSs is in continuous glucose monitoring (CGM), particularly for diabetes management. These sensors can measure glucose levels in blood or interstitial fluid, providing real-time data that aid in maintaining optimal glycaemic control [74,75,76,77]. pH monitoring is used in assessing metabolic conditions and the body’s acid–base balance, which is vital in some critical care settings and surgical procedures [78,79,80,81]. Additionally, fibre optic sensors are used for blood component detection [75], to detect specific proteins [82,83], enzymes [76,84,85], and hormones [35,86,87], aiding in the diagnosis and monitoring of various diseases, such as cancer [88,89,90] and hormonal imbalances [36,91]. These sensors can be functionalized to detect biomarkers at the molecular level, enabling early disease detection and the tracking of treatment efficacy. By measuring these biochemical parameters, fibre optic sensors provide data that present real time monitoring opportunities, enhancing diagnostic accuracy and treatment monitoring, which are directly related to biochemical measurands [36,92,93].

FOSs are adept at detecting various substances in both gas and liquid phases. In the gas phase, FOSs can detect a wide range of gases, including oxygen, carbon dioxide, and Volatile Organic Compounds (VOCs) [94,95,96,97] with high sensitivity, useful in respiratory monitoring [65,98] and detecting gases in environmental health studies. In the liquid phase, FOSs are widely used for measuring biochemical substances, such as glucose, electrolytes, and pH levels in bodily fluids like blood, urine, and saliva [91,99,100,101]. This capability is essential for continuous glucose monitoring in diabetic patients and assessing kidney function or urinary protein [100,102,103]. Accurate, real-time measurements can be achieved using different sensing mechanisms, based on different responsive materials. These sensors also provide access to a vast range of use cases, as described in Table 2 [28,104,105]. Table 2 provides an overview of recently developed FOSs designed for pH measurement and glucose detection, as well as the detection of certain cancer biomarkers and hormones, highlighting their fibre type and detection range with the responsive material.

**Figure 5:**
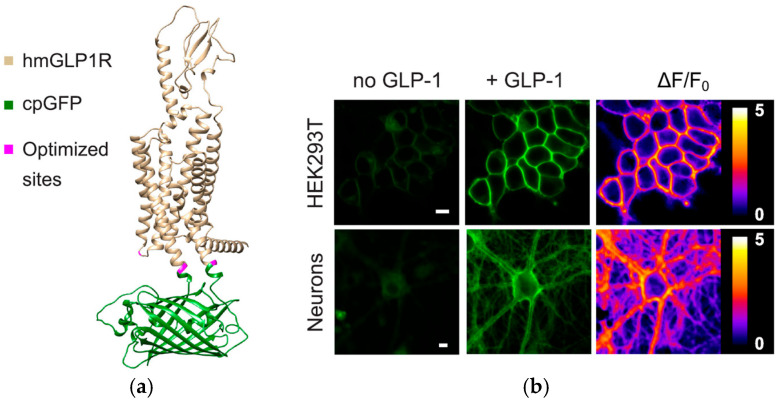
The development and optical properties of GLPLight1 were examined using structural modelling and fluorescence imaging. (**a**) The structural model, generated using Alphafold [125], depicts the human glucagon-like peptide-1 receptor (GLP1R) in gold, the circularly permuted green fluorescent protein (cpGFP) in green, and mutagenesis target residues in magenta. (**b**) Fluorescence imaging in HEK293T cells and primary cortical neurons demonstrated an increase in fluorescence intensity. Pixel-wise ΔF/F0 images further confirmed these fluorescence changes, supporting the sensor’s effectiveness in detecting GLP-1 interactions. Image source: [124].

FOSs have also demonstrated potential in monitoring glucagon-like peptide-1 (GLP-1), a critical biomarker in glucose metabolism and insulin regulation. The ability to continuously monitor GLP-1 levels offers significant advantages in managing diabetes and other metabolic disorders. A genetically encoded sensor (GLPLight1) has been developed by engineering a circularly permuted green fluorescent protein into the human GLP-1 receptor (GLP1R), as shown in Figure 5. This sensor accurately detects receptor conformational activation in response to pharmacological ligands, as indicated by its fluorescence signal [123,124].

**Figure 6:**
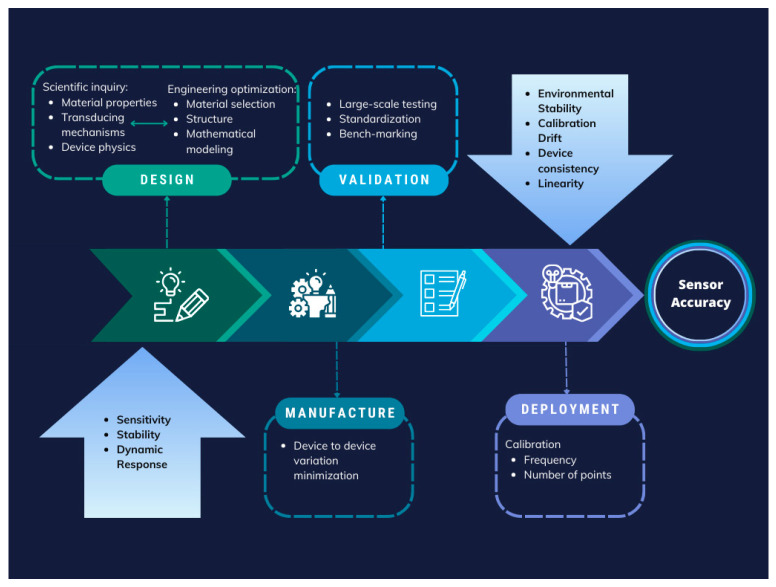
Step graph of an approach to sensor accuracy assurance during the design, manufacturing, validation, and deployment of sensor technology; factors to consider are highlighted in blue in the first and the last steps. Adopted with permission from [126]. Copyright 2025 American Chemical Society.

Ensuring accurate parameter values is paramount in sensor technology, particularly in the case of fibre optic sensors for biomedical applications. A comprehensive approach to achieving high accuracy must span the entire lifecycle of sensor development and deployment, as shown in the example step graph of Figure 6. This entails fundamental research into materials during the design phase to comprehend material properties, transduction mechanisms, and device physics, ultimately leading to optimized materials and device structures. Iteration between scientific inquiry and engineering optimization enhances sensor accuracy, considering factors such as the 3S’s (size, speed, and sensitivity).

Fabricated device accuracy and consistency are pivotal in moving towards manufacturability and deployment. Additionally, large-scale validation employing standardized procedures and benchmarking against gold-standard measurements are indispensable for obtaining reliable calibration curves [126].

## 2. Challenges for FOSs in Biomedical Applications

**Figure 7:**
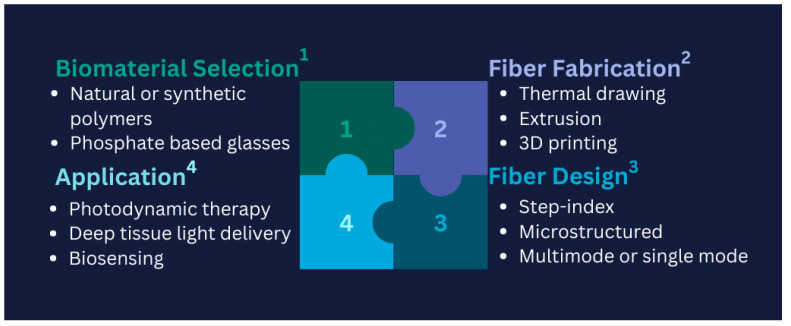
Schematic puzzle of the four significant challenges in developing biodegradable and biocompatible optical fibres; adopted idea: [127].

Developing biodegradable and biocompatible optical fibres for biomedical applications presents several key challenges. Firstly, material selection is critical, as the fibres must degrade safely in the body without releasing toxic byproducts. Natural or synthetic polymers, such as polylactic acid (PLA) or phosphate-based glasses, must be carefully evaluated for their mechanical properties and degradation rates to match specific biomedical requirements. Secondly, fabrication techniques such as thermal drawing, extrusion, or 3D printing must be optimized to ensure that the fibres maintain structural integrity while also being scalable for clinical applications. Thirdly, fibre design plays a significant role; features like microstructure or step-index profiles must balance light-guiding efficiency with mechanical flexibility and controlled biodegradability. Finally, application-specific considerations must be addressed, such as ensuring effective light delivery in photodynamic therapy or accurate biosensing under dynamic physiological conditions. PLA-based fibres have shown promise due to their adjustable degradation rates and biocompatibility. However, challenges such as mechanical fragility and inconsistent degradation in physiological environments necessitate further research. Similarly, phosphate-based glass fibres are attractive for their complete dissolution in biological fluids, but optimizing their mechanical properties without compromising their optical performance remains a significant challenge. These challenges highlight the need for continued advancements in material science and fibre design to achieve reliable, biodegradable, and biocompatible optical fibres for diverse biomedical applications [127]. Figure 7 illustrates a schematic representation of the four key challenges encountered in developing biodegradable and biocompatible optical fibres.

### 2.1. Biocompatibility

Ensuring non-toxicity for any material to be used in fabricating sensors is one of the primary challenges to achieving biocompatibility. The selection of materials needs to ensure that the FOSs are mechanically safe, non-toxic, and do not provoke an immune response when implanted in or used on the human body. This requires extensive testing to ensure that the materials do not cause inflammation, allergic reactions, or any other adverse effects [69,128]. This is generally conducted by regulating bodies, such as the USA’s Food and Drug Administration (FDA) (Section 2.5 of this article).

FOSs often require coatings to enhance biocompatibility. These coatings must be able to withstand the harsh biological environment without degrading. Ensuring the long-term stability and functionality of these coatings is crucial for the sensors’ reliable performance and regulatory approval. Furthermore, sensors often must endure various sterilization methods such as autoclaving, gamma irradiation, or chemical sterilization. A significant challenge remains to design sensors that maintain their performance characteristics post-sterilization, as these processes can sometimes compromise sensor integrity and functionality [24,129]. Table 3 presents an overview of biomaterials used in fabricating optical fibres, highlighting their base materials, advantages, and disadvantages, based on the literature.

### 2.2. Miniaturization, Durability, and Longevity

Reducing the size of fibre optic sensors without sacrificing sensitivity or accuracy is a significant ongoing challenge. Miniaturized sensors must still be capable of delivering accurate and reliable measurements [143]. Also, small-sized sensors need to be seamlessly integrated into medical devices and systems, often requiring custom design solutions [103]. This integration must ensure that the sensors do not interfere with the overall device performance and that they are easy to incorporate into existing medical infrastructure [92]. Scaling up miniaturized FOSs while maintaining quality and reliability remains a major challenge, especially for long-term biomedical applications [144,145].

Nanostructured materials have emerged as a solution to many of these challenges. The unique optical and biological properties of nanomaterials, such as size-dependent signal amplification, plasmon resonance, and enhanced charge-transfer abilities, improve the sensitivity, specificity, limit of detection, response time, and signal-to-noise ratio of fibre optic biosensors. The integration of biocompatible nanomaterials with selective bioreceptors has established a strategy for advancing FOS performance. Recent literature explores different dimensions of nanomaterials (0D, 1D, 2D, and 3D structures), although a detailed discussion is beyond the scope of this review [146,147].

Notable technologies and advancements include LSPR (Localized Surface Plasmon Resonance) fibre optic biosensing platforms [145]; THz-metasurface-mediated nano-biosensors [148]; hybrid nano-structured SPR biosensors, developed for breast and cervical cancer detection [149]; and using nanoparticles for biosensing a variety of viruses, such as SARS-CoV-2 [150]. These approaches significantly contribute to miniaturization efforts and functional enhancements. However, challenges like material stability, reproducibility, large-scale manufacturability, and environmental sustainability remain key barriers to clinical translation. Some of the innovations, such as green synthesis methods, continuous flow nanomaterial production, and advanced surface engineering techniques, are actively addressing these issues and paving the way toward the next generation of reliable, scalable, and sustainable diagnostic tools [151].

Also, based on the usage of a sensor, it could be advantageous if the sensor can be delivered inside a standard medical catheter, as this often overcomes the problem of mechanical robustness and durability [152,153,154].

### 2.3. Signal Processing, Data Integration, and Interoperability

Biological environments are often inherently noisy, e.g., external electromagnetic interference from scanning equipment (MRI and CT) can interfere with the signals detected by fibre optic sensors. If it is impossible to make the sensor immune to these sources of interference, developing advanced algorithms and signal processing techniques to filter out this noise becomes crucial for accurate measurements. The signals from FOSs often require sophisticated interpretation, especially when monitoring dynamic biological processes. This necessitates the development of advanced computational models and machine learning algorithms to analyse and interpret the data accurately [155,156,157].

Many types of medical applications require real-time data processing and feedback. Ensuring that the sensor systems can handle the computational load and provide timely, accurate information is a significant and ongoing technical challenge. FOSs must be compatible with healthcare IT systems and electronic health records (EHRs). Ensuring seamless integration and data interoperability is therefore essential for effective use in clinical settings [158,159].

Developing standardized data protocols to ensure that data from fibre optic sensors can be easily shared and interpreted across different platforms and systems is crucial. This includes ensuring sound data security and maintaining patient privacy. Finally, at this stage, providing user-friendly interfaces that allow healthcare professionals to interact with and interpret data from fibre optic sensors is important for their adoption. This involves developing intuitive software and visualization tools [92,160,161].

### 2.4. Production Cost and Manufacturing

High production costs can hinder the widespread adoption of fibre optic sensors. Developing cost-effective manufacturing processes without compromising quality and performance is crucial for making the sensors affordable [42,93]. Additionally, it may be necessary to accommodate further scaling up of production while maintaining consistency and reliability. The latter requires advanced manufacturing techniques and stringent quality control measures to ensure each sensor meets the required standards. Finally, achieving economies of scale to achieve lower costs involves improving manufacturing processes and increasing market demand and production volumes. This can also be challenging in the early stages of technology adoption [162,163].

### 2.5. Medical Standards and Regulatory Approval

Medical and/or biomedical FOSs must meet stringent regulatory standards set by organizations such as the FDA (Food and Drug Administration) and EMA (European Medicines Agency) [164,165,166,167,168,169]. This involves extensive testing to demonstrate safety, efficacy, and reliability.

Ensuring compliance with international standards for medical devices is critical. This includes adhering to ANSI (American National Standards Institute)/AAMI (Association for the Advancement of Medical Instrumentation) or ISO standards, such as AAMI/ISO 10993, ISO 13485, and AAMI TIR42: Technical Information Report (TIR), not a formal standard, but rather guidance for evaluating biocompatibility in alignment with ISO 10993, which helps manufacturers interpret biocompatibility requirements for regulatory compliance [164,170,171].

Also, regulatory frameworks governing medical devices within the European Union include the EU Medical Device Regulation (MDR) and the In Vitro Diagnostic Regulation (IVDR) [167,168,170]. Obtaining regulatory approval can be lengthy and complex, requiring a long lead time and many resources. This can delay the introduction of new fibre optic sensor technologies to the market and their wider application. Table 4 outlines the regulatory standards governing using fibre optic sensors in medical applications.

### 2.6. Ethical Considerations

When developing and deploying sensor technologies for biomedical applications, there is a range of ethical challenges that researchers and clinicians need to consider. These include ensuring user autonomy by preventing unnecessary dependence on the technology or burdening participants, and promoting beneficence by balancing societal benefit against individual risk while protecting participant privacy and dignity. Transparency is essential, as participants should be informed that they may not receive direct clinical benefit and that the research is for broader knowledge generation. Issues of justice particularly regarding equitable access, data usage, and potential commercialization must be addressed. Informed consent processes should be clear, dynamic, and include explicit examples of data collection. Researchers must communicate that participation does not equate to guaranteed well-being and foster trust through respectful engagement. Oversight mechanisms and conflict-of-interest disclosures are vital, as is ensuring that researchers understand both the technical and social impacts of these technologies, including compliance with local privacy laws [173].

FOSs offer advantages for biomedical applications, including high sensitivity, immunity to electromagnetic interference, compact size, biocompatibility, and the ability for real-time, minimally invasive monitoring. Their versatility allows for the detection of a wide range of physical, chemical, and biochemical measurands with excellent spatial resolution [174]. However, despite these strengths, FOSs also face notable disadvantages. Manufacturing complexity, especially for miniaturized and nanostructured designs, remains a major challenge. Long-term stability in harsh biological environments, signal degradation over time, the need for specialized interrogation systems, and high production costs can limit large-scale clinical deployment [3]. Moreover, achieving consistent reproducibility and meeting regulatory standards adds additional barriers to translation from laboratory prototypes to real-world medical devices [175,176]. Table 5 summarizes the key challenges involved in developing FOSs for use in biomedical applications.

## 3. Future Perspectives

Looking ahead, the integration of advanced materials, green nanotechnology, and hybrid photonic platforms is expected to address many of the current limitations of FOSs. The convergence of fibre optic sensing with machine learning [177], AI technologies [178,179], IoT (Internet of Things) technologies [180], robotics [181], tactile sensing [182], and wearable devices [183] opens new possibilities for personalized and predictive healthcare. Future research will likely focus on improving sensor robustness, achieving scalable manufacturing, enhancing environmental sustainability, and ensuring ethical and regulatory compliance for clinical applications. Ultimately, fibre optic biosensors are poised to become a critical part of next-generation diagnostics, offering real-time insights and improving outcomes across a wide range of medical fields [176,184].

## 4. Conclusions

FOSs have been transformative in healthcare, offering high accuracy and versatility in monitoring physiological and biochemical parameters, including temperature, pressure, strain, and biochemical markers, enhancing diagnostics and patient outcomes. However, the widespread adoption of FOS technology in healthcare faces several critical challenges, including biocompatibility, miniaturization, durability, and robust signal processing. Addressing these challenges requires interdisciplinary collaboration across material science, engineering, and medical practice to develop reliable, scalable, and clinically viable sensor systems.

Innovative advancements in biocompatible materials, fabrication techniques, and signal processing algorithms continue to push the boundaries of what FOSs can achieve in medicine. Standardization and regulatory approval remain key hurdles that must be overcome to facilitate their transition from research laboratories to commercial medical devices. Throughout the process of addressing all challenges, ethical considerations must also be contemplated. Future research efforts should focus on enhancing sensor integration within existing medical systems, improving long-term reliability and stability, and developing AI-driven analytical methods for accurate data interpretation for personalized and predictive healthcare.

In overcoming these challenges, fibre optic sensors have the potential to revolutionize biomedical sensing, paving the way for more precise diagnostics, personalized treatment plans, and improved patient outcomes. As the field advances, the synergy between optical sensor technology and emerging biomedical innovations will shape the future of healthcare, making real-time, minimally invasive monitoring an integral part of medical practice.

## Figures and Tables

**Table 2:** Overview of recent FOSs for pH, glucose, cancer biomarker, and hormone measurement/detection.

Sensing Application	Responsive Material with Fibre Type	Detection Range	Sensitivity	LOD (Limit of Detection)	Ref.
pH	PANi with TFBG	2–12	minimum of 30 pm/pH maximum of 82 pm/pH	-	[106]
	PAAm hydrogel with SPR	8–10	13 nm/pH at	-	[107]
	gold nanoparticle-functionalized fibre optic probes with FPI	2–12	1.95 nm/pH	-	[80]
	Hydrogel + polymer microarrays with miniature optical fibre	5.5–8	mean precision of 0.10 pH units	-	[79]
glucose	GO/GOD with LPFG	0–8 mM	∼0.24 nm/mM	-	[108]
	GOD with multimode microfibre	0.0–166.67 mM	1.74 nm/(mg/mL)	-	[109]
	3-APBA with LDOF	0–50 mM	2.6 μWmM^−1^		[74]
	GO with LPFG	0∼1 wt%	6.229 dB/wt%		[110]
	GO/GOD with PCF	10 g/L to 70 g/L	-	-	[111]
	SPR with Microsphere optical fibre	0–200 mg/dL	0.1688 nm/(mg/dL)	4 mg/dL	[112]
	Gold nanoparticles (AuNPs) and LSPR with TOF	1.328–1.393 (5–45 wt%)	For bare TOF: 1265%/RIU For AuNP-decorated TOF: 2032%/RIU	-	[113]
	GO/GOD with PS-LPFG inscribed on high-birefringence fibre (HBF)	5–25 mM	∼20.8 pm/mM	-	[114]
	Gold-coated plasmonic layer with PCF	Not specified	2500 nm/RIU (wavelength), 152 RIU^−1^ (amplitude)	-	[115]
	SPR with enzymatic reaction	0–400 mg/d	3.10 pm/(mg/dL)	-	[76]
	gold nanoparticle-functionalized fibre optic probes with FPI	1 μM–1 M	3.25 nm/mM	-	[80]
cancer biomarkers	Tapered fibre optic interferometer cascaded with FBG for HER2 protein	-	-	2 ng/mL	[90]
	FOSs catheter embedded for CD44 protein	-	-	4.68 aM	[116]
	Ti_3_C_2_-supported gold nanorod hybrid nanointerfaces with microfibre, integrated with hybrid nanointerfaces	-	-	13.8 zM (buffer), 0.19 aM (30% serum)	[117]
hormones	Oestrogen receptor on gold-coated polystyrene with Spoon-shaped SPR	-	-	0.1 pM	[91]
	Thin gold layer with 7-core fibre + SMF for Insulin	-	-	10^−8^ g/mL	[118]
	Oestrogen receptor with gold-coated tilted fibre Bragg grating (TFBG)	-	-	1.5 × 10^−3^ ng/mL	[119]
	Anti-cortisol antibody on AuPd-coated with SPR on plastic optical fibre (POF) for cortisol	0.005–10 ng/mL	-	1 pg/mL	[120]
	Anti-cortisol antibody on gold-coated D-shaped SPR for cortisol	0.01–100 ng/mL	0.65 ± 0.02 nm/log(ng/mL)	1.46 ng/mL	[121]
	Gold nanoparticles with FOS microfluidic channel for thyroglobulin (Tg)	-	-	93.11 fg/mL	[122]

**Table 3:** Overview of biomaterials utilized in optical fibre fabrication [127,130].

Material Type	Material Example	Advantages	Disadvantages	Refs.
Natural	Proteins: silk Polysaccharides: alginate, cellulose, agarose, chitosan, gelatine	biocompatibility and biodegradability	limited design flexibility, restricted availability and quantity, batch-to-batch variability, low mechanical strength, and potential immunogenicity	[131,132,133,134,135]
Synthetic	Hydrogels: Polyethylene Glycol (PEG), Pluronic (Poloxamer) Citrate-based elastomers: poly (Octamethylene Citrate) (POC), poly (Octamethylene Maleate Citrate) (POMC), Polymer-Based: Polyvinyl Chloride (PVC), SU-8 (Negative Photoresist Polymer), Poly (L-Lactic Acid) (PLLA), Poly (D, L-Lactic Acid) (PDLLA), Poly (L-Lactic-Co-Glycolic Acid) (PLGA), Poly (D,L-Lactic-Co-Glycolic Acid) (PDLGA), Poly-ε-Caprolactone (PCL) Inorganic materials: Calcium–Phosphate Glass (PGs) Silicon-Based Materials: Silicon, Polydimethylsiloxane (PDMS)	adaptable and flexible structure, tunable biodegradability, and customizable physical, mechanical, and chemical characteristics	biocompatibility should be verified and the rigidness and brittleness of glass should be confirmed	[22,127,136,137,138,139]
Hybrid Biomaterials (Natural and Synthetic)	Chitosan and Polystyrene Membranes/PAA Silk Fibroin Film, Agarose hydrogel (AG) with gold nanoparticles (AuNPs)	biocompatibility, mechanical strength and tunable properties for PAA, controlled permeability, chemical resistance	limited flexibility, surface modification required for some, degradation issues, Processing complexity, for AuNPs, agglomeration of AuNPs, and limited long-term stability	[133,134,140,141,142]

**Table 4:** Regulatory standards for fibre optic sensors in medical applications [164,165,166,167,168,169,170,171,172].

Regulatory Body	Standard/Guideline	Scope and Relevance to FOSs
FDA (USA)	FDA Medical Device Approval Process	Safety, efficacy, and reliability assessments ensure FOSs meet regulatory requirements before market approval.
NCAs (EU)	Medical Device Regulation (MDR) and In Vitro Diagnostic Regulation (IVDR)	Regulation of general medical devices in the EU governs their safety and performance.
ISO	ISO 13485/ISO 10993	Quality management system for medical devices/Biocompatibility evaluation of medical devices.
AAMI/ANSI	AAMI TIR42	Guidance on biocompatibility evaluation, which supports compliance with ISO 10993 for medical FOSs.

**Table 5:** Challenges in developing FOSs for biomedical applications.

Challenge	Key Considerations	Refs.
Biocompatibility	- Ensuring non-toxicity, immune safety, and mechanical stability of materials - Coatings for biocompatibility	[127,128]
Miniaturization and Nanomaterials	- Maintaining accuracy in miniaturized sensors - Integration into medical devices - Advancements in nanomaterials for improved sensitivity - Long-term sensor reliability	[145,146]
Signal Processing and Data Integration	Fast and accurate data processing - Ensuring seamless system integration with medical devices - Real-time data transmission and processing	[158,159]
Production Cost and Manufacturing	- Balancing cost-effectiveness with high quality - Ensuring scalability in production	[93,161]
Medical Standards and Regulatory Approval	- Adhering to FDA and other regulatory body requirements - Ensuring sensor stability after sterilization - Ensuring human safety testing and approval	[164,165,167,170]
Ethical Considerations	- Ensuring patient privacy, safety, and secure data transmission - Obtaining informed consent and preventing misuse of health data	[173]

## Abbreviations

The following abbreviations are used in this manuscript:

FOSs	Fibre Optic Sensors (FOSs)
POSs	Polymer-based optical sensors
OF	Optical Fibre
SPR	Surface Plasmon Resonance
POFs	Polymer Optical Fibres
FBGs	Fibre Bragg Gratings
OCT	Optical Coherence Tomography
IOP	Intraocular Pressure
PCF	Photonic Crystal Fibre
SMF	Single-mode Fibre
FPI	Fabry–Pérot Interferometer
MMF	Multi-mode Fibre
MEMs	Micro-Electromechanical Systems
LSPR	Localized Surface Plasmon Resonance
LMR	Lossy Mode Resonance
HST	Hollow Silica Tube
PID	Proportional Integral Derivative
PANi	Polyaniline
TFBG	Tilted Fibre Bragg Grating
PAAm	Polyacrylamide
GO	Graphene Oxide
GOD	Glucose Oxidase
LPFG	Long-period Fibre Grating
TOFI	Tapered Optical Fibre Interferometer
3-APBA	3-Aminophenylboronic Acid
LDOF	Lossy Dielectric Optical Fibre
HBF	High-birefringence fibre
PLA	Polylactic acid
FDA	Food and Drug Administration
PEG	Polyethylene Glycol
POC	Poly (Octamethylene Citrate)
POMC	Poly (Octamethylene Maleate Citrate)
PVC	Polyvinyl Chloride
SU-8	Negative Photoresist Polymer
PLLA	Poly (L-Lactic Acid)
PDLLA	Poly (D, L-Lactic Acid)
PLGA	Poly (L-Lactic-Co-Glycolic Acid)
PDLGA	Poly (D, L-Lactic-Co-Glycolic Acid)
PCL	Poly (ε-Caprolactone)
PGs	Phosphate Glass
PDMS	Polydimethylsiloxane
PAA	Polyacrylic Acid
AG	Agarose Hydrogel
AuNPs	Gold Nanoparticles
MRI	Magnetic Resonance Imaging
CT	Computed Tomography
EHRs	Electronic Health Records
NCAs	National Competent Authorities
ANSI	American National Standards Institute
AAMI	Association for the Advancement of Medical Instrumentation
TIR	Technical Information Report
MDR	Medical Devices Regulation
IVDR	In Vitro Diagnostic Regulation
